# Leadership for systems change: Researcher practices for enhancing research impact in the prevention of chronic disease

**DOI:** 10.3389/fpubh.2022.1045001

**Published:** 2022-12-06

**Authors:** Melanie Pescud, Lucie Rychetnik, Steven Allender, Michelle J. Irving, Eloise Howse, Harry Rutter, Ray Ison, Therese Riley, Sharon Friel, Diane T. Finegood

**Affiliations:** ^1^Menzies Centre for Health Governance, School of Regulation and Global Governance (RegNet), Australian National University, Canberra, ACT, Australia; ^2^The Australian Prevention Partnership Centre, The Sax Institute, Glebe, NSW, Australia; ^3^School of Public Health, Faculty of Medicine and Health, University of Sydney, Camperdown, NSW, Australia; ^4^School of Health and Social Development, Faculty of Health, Deakin University, Geelong, VIC, Australia; ^5^Centre for Evidence and Implementation, Carlton, VIC, Australia; ^6^Department of Social and Policy Sciences, University of Bath, Bath, United Kingdom; ^7^School of Engineering and Innovation, Faculty of Science, Technology, Engineering and Mathematics, The Open University, Milton Keynes, United Kingdom; ^8^Therese Riley Consulting, Melbourne, VIC, Australia; ^9^Mitchell Institute, Victoria University, Melbourne, VIC, Australia; ^10^Morris J. Wosk Centre for Dialogue, Simon Fraser University, Vancouver, BC, Canada

**Keywords:** system, leadership, change, prevention research, chronic disease, research impact

## Abstract

**Introduction:**

Strengthening systems for chronic disease prevention is essential. Leadership for systems change is an important key to strengthening systems. Leadership in prevention research for supporting systems change remains a relatively abstract concept and there is limited empirical information about the leadership practices of prevention research teams when viewed through a complexity lens. In this paper we examine and describe some systems leadership practices for creating change through prevention research, as identified in a series of six case studies.

**Methods:**

A qualitative approach incorporating semi-structured interviews, participant observation, and document review was used to facilitate an in-depth investigation of the research topic.

**Results:**

Several researcher practices for enhancing research impact in the prevention of chronic disease were distilled from the data pertaining to how they sought to create change. These included persuasive communication, compassion and deep listening, reflective practice, and embedding themselves within the systems they sought to change.

**Discussion:**

The findings provide insights that may assist prevention researchers and other practitioners dedicated to creating change in chronic disease prevention.

## Introduction

Chronic disease presents a considerable burden on population health at both local and global levels (1–3). Key elements to creating a stronger purpose-driven, chronic disease prevention system include focusing on health equity, dedicating sufficient resources and information, improving collaboration and implementation capacities, actively applying a systemic paradigm, and fostering leadership for on-going, adaptive systems change (4).

The goals of prevention research are many, but prevention research ultimately seeks to inform, support, and have impact on preventive action (i.e., interventions, programs, policies, and large-scale institutional and organizational change). The absence of a co-owned, purposeful prevention research system is often a limitation in supporting the impact of that research. The amelioration of this requires what we refer to in this paper as “systems leadership.” Yet systems leadership in prevention research for supporting systems change remains a relatively abstract and opaque concept, and there is limited empirical information about the leadership practices of prevention research teams when viewed through a systems theoretical and complexity lens.

Our previous work has examined the importance in prevention research of integrating a systemic lens with an implementation focus linked to a theory of systems change. We have also explored the complementary interplay between systemic and systematic approaches in prevention research (5, 6). The broader practices of systems leadership have been described for other settings [e.g., (7–9)]. These include commitment to the viability of a whole system, deep listening, ability to see reality through the eyes of people very different from oneself, and openness and commitment to ongoing growth and learning (7, 8). It is also recommended that such practices be distributed throughout a team, organization, or larger group to create sustainable change and reduce the reliance on a single “heroic” leader (10, 11). These practices within a team provide an antidote to traditional ideals of leadership that have had minimal impact on solving complex problems (12).

In this paper we present practices described in six prevention research case studies (5, 6) to propose important elements of leadership for systems change. This analysis of interviews with researchers focused on addressing complexity in chronic disease prevention and identified recurring patterns in the qualities, perspectives, framing, and practices that were associated with a focus on creating systems change within the prevention system.

## Materials and methods

### Research design

We conducted a comparative case study using qualitative methods with the overarching goal of exploring how chronic disease prevention researchers address complexity in prevention research [see (6) for a full description of the study methods overall for the larger study]. A qualitative approach incorporating semi-structured interviews, participant observation, and document review was chosen to facilitate an in-depth investigation of the research topic (13). Ethics approval was granted by the Australian National University human research ethics committee–ref no 2019/653.

### Recruitment

#### Case selection and participants

A purposive sample of six prevention research case studies was chosen. Cases had either been fully or partially funded by the Prevention Center or affiliated with the Center. Given the focus on complexity, the following criteria were used to recruit case studies: use of systems thinking (implicitly or explicitly), application of systems approaches and systems science methods/tools to a component of the work, application of traditional methods/tools to a component of the work, and or use of systems theories. The six cases focused on studies of liveability (making communities healthier places to live), rating and benchmarking the food environment, community-based childhood obesity prevention, intersectoral action to address inequities in healthy eating, embedding prevention research within health systems, and using dynamic simulation modeling as a decision-support tool for addressing childhood obesity [see (6)]. Each case study operated within its own system, which is generally defined as an interconnected set of biological, behavioral, social, environmental, and economic factors. Our focus was on cases where these either helped or hindered chronic disease prevention efforts, and influenced their change making focus.

Participants held positions in academic, government, and the not-for-profit sectors, and a handful held cross-sectoral roles; for example, working in both government and academia or academia and the not-for-profit sector. Participants' roles in the system, the types of change they were involved in (e.g., influencing policy, mindsets, and community actions), and their practices all appeared to operate in a complex and interdependent manner which enabled them to successfully influence or produce change within the systems in which they worked.

#### Data collection

Case study participants were recruited *via* a personalized email invitation with an information letter and consent form attached. Interviews were held over the phone or *via* zoom video, digitally recorded with permission, and transcribed verbatim style by a professional transcription service.

Data comprised two sets of qualitative interviews scheduled four to six weeks apart and associated project materials (available online). In total, 29 interviews were conducted with 16 study participants. For the document review, we collated project information from relevant websites comprising peer-reviewed publications and findings briefs. This information served to complement the interview transcripts, thus providing a more comprehensive picture of the research work relating to each case study.

The first set of interviews served to introduce participants to the study purpose, explore the background and context of their research, and deeply understand how they carried out their work. Language associated with systems theory, such as feedback loops and stocks and flows, were avoided by the interviewer unless referred to and used by the participants. In the second set of interviews, the goal was to take a deeper look into how research teams and individual participants addressed complexity within their work. A systemic lens was applied to the interviews and guided by an adapted version of Foster-Fishman et al.'s (14) framework for understanding and changing systems. We chose this framework for its theoretical depth and breadth covering literature on systems thinking, community change, organizational change, as well as its successful previous application (14, 15). Key areas for exploration within the framework included systems norms, systems resources, systems regulations, and systems interdependencies (14). To suit the prevention research context of our study, we adapted the sets of questions within each of the key areas to elicit deeper insights into how prevention researchers responded to complexity in their work.

### Data analysis

Data analysis occurred in a staged manner both within and across the case studies focusing on identifying the many ways in which teams addressed complexity. Further analysis was then conducted to explore leadership for systems change for the present study. Both inductive and deductive processes were employed throughout whereby Foster-Fishman et al.'s (14) framework was used to inform coding categories and theoretical sensitivity; Glaser and Strauss' (16) grounded theory principles informed the development of new codes and 15 reflexive thematic analysis informed the approach to coding. More details of the staged analysis can be found in Pescud et al. (6).

All interview transcripts were coded individually by a minimum of two authors (MP, LR, and or MI). The coding process was carried out using the Microsoft Word comment function and followed by memo writing within each transcript. Deductive codes were informed by a combination of literature on systems approaches, frameworks, models, and tools including the Intervention Level Framework, complex adaptive systems characteristics, community-based systems dynamics work, and systems change (14, 17–19).

Next, a structured framework analysis was carried out in Google Sheets to allow for comparisons across the case studies and within categories. The framework was devised by creating broad categories comprised of several combined codes informed by the systems literature (14, 17–19) as well as the inductive codes (16, 20).

While a central part of the analytical process was exploring the broad research question of how prevention researchers address complexity in their work [see (6)], an important theme generated from the data analysis process related to leadership and its relationship with change making. This included the dependence of systems change on leadership capabilities that support change making within complex systems as well as leadership structures. We decided to explore the recurring theme of creating systems change by focusing in on a smaller number of categories within the framework as this was an important element of all interviews.

### Research reflexivity

Our core team were LR and MP; at the time of the project, LR was Co-Director of the Prevention Center, while MP was a Senior Research Fellow at the Australian National University. Throughout the study, our team of co-authors contributed to our ideas, analyses, and writing. This occurred *via* in-person and virtual conversations and written feedback within draft manuscripts. We also drew from the systems literature to inform our work and analytical processes (14, 17–19, 21, 22). We acknowledge that the data we collected and interpretations made are informed by our team's worldviews, assumptions, beliefs, and experiences in the area, the research question and study design, and the overall goals of the Prevention Center to explore ways to apply systems thinking and systems science to the study of chronic disease prevention.

## Results

Several researcher practices were distilled from the data pertaining to how they sought to enhance their research impact for the prevention of chronic disease. These practices included persuasive communication, compassion and deep listening, reflective practice, and embedding themselves within the systems they seek to influence. The key findings center on what participants reported to be the core drivers and motivations for their work and the way in which they described their approach in practice to the goal of creating change. The leadership practices reported are a combination of concepts that the participants described of themselves and what we as researchers observed through our analysis.

### Persuasive communication

Being able to communicate in a persuasive manner was a capability and practice reported by participants for influencing change. Persuasive communication was fostered through various practices including careful use of language, the ability to foster shared values and understanding, the rigorous use of research evidence, the ability to evoke the collective community, and having or creating legitimacy and authority.

#### Careful use of language

Participants spoke about the importance of adapting their use of language and style of communication to fit the needs of the various stakeholders with which they interacted. They spoke at length about the need to clearly communicate, which in many cases meant refraining from using jargon and learning to speak the language of different communities, be they policymakers, academics, or local community members. In some cases, however, the use of jargon was considered beneficial and was used as a way of ensuring all stakeholders within a room were on the “same page,” thus enabling a shared understanding to be established when it came to the technical use of systems science terms. For example, when working with epidemiologists to build a dynamic systems model, content was explained in terms of prevalence, incidence, and duration, whereas when working with people with a lived experience of mental health issues researchers explained things in terms of pathways and how people flowed through systems, and where they went to get what they needed. Making the language relevant to different ways of thinking meant that the various stakeholders could critique the work in a meaningful way. In a dynamic simulation modeling project, the lead researcher described the importance of common language:

*We need a common language to help everyone feel they're on the same level and because system dynamics language is in talking about stocks and flows and pathways, it's new to everyone. It kind of levels the playing field a bit, which is a good thing in that environment*.

Communication also went beyond verbal interaction to include physical attire and served to either break down power structures or reinforce them where appropriate to ensure communities and stakeholders were engaged. For example, one researcher reported that they would deliberately dress formally as an authority figure or casually in order to convey an easy-going attitude depending on who they were meeting. This was based on feedback from community leaders on what would work best to serve the community's needs.

#### Shared values and understanding

Participants described how important it was to link their research to a set of values that underpinned their work which also enhanced stakeholders' trust in their work and gave weight to the reasons why they were driven to work in their chosen area. A “sense of living with principles” was something that was highlighted by participants as being key to their values-driven work. In alignment with their values, they reported consciously choosing a path that enabled them to achieve their goals. In those cases where values did not necessarily align with those they collaborated with, they ensured shared understanding was achieved so that all parties' needs were met through various means. For example, when working with a property developer, one researcher was keenly aware of the developer's goals to make money, while at the same time being part of the affordable housing solution. By recognizing that there was a shared value of helping humanity, they were able to create a working relationship based on mutual understanding and respect.

*I can't let my values of equity, and fairness, get in the way of knowing that they also want to make money. And I have no problem with that, as long as they're not screwing over other people… So, we share a common value of housing for all…He's just horrified that the largest, fastest growing homeless sector in Australia are women over 50. He said it's embarrassing, it's terrible, and we've got to do something about that. So, our basic value is humanity, and doing the right thing by humanity*.

#### Rigorous use of research evidence

The ability to convey the merits of participants' programs of research and the methods they used was a key aspect of persuasive communication with stakeholders which also served to contribute to the development of trust between parties. Using rigorous research evidence and drawing upon the work of others was a necessary component for both gaining community support and informing their programs of work. In the case of those using innovative new methods within public health, such as established tools from system science, they spoke to the long and robust histories of use in other fields, in some cases spanning two hundred years of science.

*My broader vision has always been to use these methods that have been used for decades in infectious disease epidemiology to get them used in the broader public health sphere and the social and prevention agenda…Very rarely [have] the academics involved had any understanding of what this was, and the policymakers hadn't seen it before… while there was a long tradition in infectious disease in this work, there was not very much at all in chronic disease prevention*.

#### Evoking the collective

Participants evoked a sense of the collective when they described their work. Their work was rarely described in terms of their individual needs; on the contrary, their work was carried out within teams for the purpose of benefiting the wider community. They saw themselves as playing a leadership role within their teams and the communities within which they worked and influenced, but they did not emphasize any individualistic or personal needs and goals that weren't also linked with the collective. Further, the successful actions taken to achieve their goals were attributed to teams and communities working in collaboration, rather than being attributed to individuals. This aspect of communication also served to build trust as demonstrated in this quote from a lead of several community-based projects.

*Scientists are all trained to think that we're going to develop a tablet that solves the disease and we're going to do the study that's an RCT with 30 people in one arm and 30 people in the other, who all take the tablet at 10 am and no longer have Malaria and I'll get my Nobel Prize and get a building named after me. And that is never going to happen if you're going to solve complex problems because as soon as it works you shouldn't be in the photo, let alone have your name on it. Those wins belong to the people who deliver them. Our job is to help them find the way to do that*.

Participants reported taking responsibility for their role within the systems in which they sought to influence and acted from a place of strong personal agency. From this position, the goal was for others to see themselves within the system too, and act from a place of agency for the benefit of the collective. Of importance is the seniority of study participants; many held associate professor, full professor, management, and or director positions, which under their positions meant they had the ability, power, and status to act with influence within systems.

#### Legitimacy and authority

In order to create change, participants explained that it was important to leverage, build, and demonstrate legitimacy and authority. Prior to being able to demonstrate it, however, it was necessary to align oneself with those who already had legitimacy and authority because they were established in their careers and had gained respect within their fields, as demonstrated in the following quote:

*He was the co-facilitator of the first modeling projects and he was deeply involved in those first applications. And since then, has been advocating strongly for the approach, to the point where all these people come to me and they say, ”He said I needed to speak to you urgently about this approach that you're doing.“ So, he's great because he's well respected and he wears multiple hats for policy and academia and he saw the benefits on both sides*.

Mentoring was an important aspect related to legitimacy and authority. Participants actively sought opportunities to be mentored by senior colleagues who embodied the traits they wanted to develop. These included learning how to work with the media, advocate for policy change, and facilitate community change-making workshops. Eventually, participants demonstrated legitimacy and authority themselves which translated to having greater leverage and capacity to influence and create change. Having been personally mentored also created an obligation and desire to mentor others as a way of paying forward the benefits they had been afforded through receiving mentoring themselves, as demonstrated by this quote from one researcher:

*He's been my mentor and I wrote a list of everything he's done for me and he said to me, ”I'm not dead.“ I said, ”I know you're not dead,“ and I only wrote three pages but they're just all the things you've done for me and I really appreciate it… So that's where mentors are so important, and if anyone ever wants to talk to me, anyone young, I always give them an hour, always, and I would do whatever I can*.

### Compassion and deep listening

Compassion was another capability and practice reported by participants for influencing change within systems. Compassion was closely related to deep listening and the development and maintenance of trust. Participants articulated the importance of being compassionate as a way of enhancing change efforts. They were keenly interested in the experiences of those living with conditions that lead to chronic disease. As well as gaining an in-depth understanding of community members' perspectives and experiences, they also sought to step into the shoes of the policymakers they aimed to influence. A key question they often asked was ”what do you need?,” thus, they actively took the time to imagine and hear what it was like to walk in another's shoes. A skill that enhanced their practice of compassion was deep listening without judgment and a sincere desire to learn. Because of this ability, they could act to address the needs of their community stakeholders and collaborators in their many and varied forms. Participants also shared that the practice of asking what was needed was key to establishing trust. Researchers were, however, able to ask what people's needs were because they held positions of power that enabled them to act upon people's needs.

*The target population, you look at them as a living being and you are thinking all the time about their behavior. So, we're asking, why doesn't a midwife ask this question of a pregnant woman in antenatal? And then we talk to them. And you just sit and listen and that's where you have the rich conversation. But she doesn't believe it and she doesn't have the skills or she's so pressed for time. And you can look at the literature and they are all common barriers as to why a clinician doesn't ask. But if you go and listen to it in the flesh, you say, well I'm going to design my program, my training in a way that speaks to what that woman said, not what the literature says, time is a barrier. So, as soon as you make something abstract, you disconnect it from the human. You should never lose sight of the humanity of the person you are seeking to change. So, you should be engaged with them, you should be meeting with them*.

### Reflective practice

Engaging in ongoing reflective practice was another capability and practice reported by participants and observed through the analysis process for influencing change within systems. Reflective practice influenced deep listening which contributed to intellectual humility which in turn fed back into the practice of reflective practice. Each of these aspects fed into capacity building efforts within both research teams and in community stakeholders that facilitated the empowerment of communities.

Many of the participants were renowned experts in their fields of research in terms of content knowledge as well as methodological approaches. While they confidently stood by their work and its high quality, participants consistently demonstrated openness and receptivity to other ways of thinking and working. There was a recognition that there was no one way to create change. Intellectual humility was also present which was underpinned by personal practices, and in some cases, professional practices, of self or group reflection. An orientation toward continual learning and evolution of practice was fostered through reflection practices (both formal and informal) and deep listening.

*I've learnt so much from these people and I've been grateful that they've been willing to help me from the beginning when I was very naïve, I didn't know anything at all. But I know a lot now, and I've known a lot from doing, and listening, and reading…I learn from them as much as they learn from me. And that's why a lot of our stuff gets picked up because I'm listening*.

*We build feedback into everything we do. So, to give you an example of that–if we were running a group model building session in a community, we'd have a couple of people from the last community and a couple of people from the next community in the room. We'd ask them to contribute in some way to the session, and then as soon as everybody's left the room regardless of the team that's there–we sit in a circle and say, “Okay, what went well? What can we improve on? What didn't feel right?” And we do it from least experienced to most experienced to break down power structures*.

### Embedding within systems

Participants reported being very deliberate about the extent to which they embedded themselves within the systems they sought to influence or change. It appeared that the use of compassion supported by deep listening enabled them to deepen their rapport and trust within relationships. This was a means by which to move closer to the inside of the “tent,” therefore being more influential. In one institution that deliberately took an embedded approach, researchers sat in the same physical space as doctors, nurses, and patients. The purpose of this was to encourage stakeholder engagement. In addition, participants spoke about the need to foster strong relationships as a way of embedding themselves within a system or systems without the need to be physically present. They also mentioned using governance arrangements which meant that structurally, they had a defined role inside of a system in which they could create change through community empowerment.

*I want my work to make a difference. So, I get in there, boots and all, but that's about engagement, it's about relating to your target audience as humans and then you design your interventions which relate to them, then you design your interventions to relate to the environmental context of them of where they fit*.

*Everyone can be caring, but it's actually about engaging with people. I sit and listen to people and find those gems about why they are doing something or why they are not doing something*.

It appeared that governance structures combined with capacity building efforts worked to generate community empowerment which was key to systems change. Participants discussed the need to build capacity with policy stakeholders and partners as well as members of the lay community and the general public. By building capacity within these groups of collaborators, which was often facilitated by embedding within systems, participants were able to empower them to think and act in ways that ensured they thought and acted from a systemic perspective. From this place, they could ask appropriate questions to work toward achieving useful solutions to the problems they sought to solve. They were also upskilled in some instances with the use of software to better understand the systems in which they lived and worked and how to take effective action across the system to move toward transformative change.

*So, the relationship is ongoing, but the relationship is adaptive as well because it's not a project-defined relationship. It's a mutual interest in tackling complex problems using system science. So, I reckon that's probably actually a massive difference because we're pigeonholed into a project starts, the project ends, evaluate project paradigm, and if what you're looking for is inter-generational change, then that's not going to happen in a three-year stint*.

*We measured readiness to change and community capacity and they were clearly drivers of the results we were seeing and so we're not just building capacity outside of our four walls, we're building capacity within our teams as well*.

## Discussion

In this paper, we have drawn out the range of systems leadership practices present within a sample of prevention researchers, in many cases linking those practices to change making successes or research impact. This is the first time, in the Australian prevention research context, this grouping of practices for systems leadership to encourage systems change has been examined. The findings provide insights that may assist prevention researchers and other professionals dedicated to creating change in the chronic disease prevention space. While the findings are centered on the Australian context, they may have relevance internationally given the growing focus within academia to place more emphasis on research impact and the processes through which impact can be generated by researchers [e.g., (23)].

The findings from this study contribute to the existing body of systems leadership literature that demonstrates the need for those seeking to create change to hold a focus on the system as a whole, to practice deep listening, to practice compassion, and to step into the shoes of another, and engage in regular reflective practices to deepen their ongoing growth and learning potential (7, 8). Other systems leadership work in public health has centered upon the need to create a compelling call to action, a coalition of the willing, and a culture fueled by strong relationships, curiosity, and a deep understanding of the system of interest (24).

To effectively create change within complex settings, the focus is best centered on creating diverse teams, exploring various opportunities for interaction and the development of a collective mind, and shifting to sense-making from decision making especially when the trajectory of a system is unknowable (25). Systems leaders are not expected to have all of the answers when problem-solving; instead, they work through a process of co-production by engaging others to ask key and pertinent questions that allow for shared decision making and co-design of solutions (26, 27). Fawkes outlines some key propositions of systems leadership for systems change in the chronic disease prevention space, namely that leaders must enable dialogue; foster connections and promote innovation; allow and encourage leadership to occur as part of both formal and informal roles; respect various types of leadership including 'servant' or quiet styles; and work toward disrupting the status quo by creating a culture of ongoing learning and growth and acknowledging uncertainty within systems.

Oliver and Cairney's systematic review (2019) explored how academics can most effectively influence policy change. They distilled eight main recommendations, all of which align with the leadership practices within our sample: (1) conduct high-quality research, (2) ensure research is relevant and accessible, (3) develop an understanding of policy processes, (4) routinely engage with policymakers humbly and flexibly, (5) make the decision to be either an advocate for particular issues or a knowledge broker, (6) build and nurture long term relationships with policymakers, (7) act like an entrepreneur or find a mentor who is, and (8) engage in ongoing reflection practices to ascertain what is working, what is not, and in doing so, being able to course correct. Our study adds to this list with the inclusion of deep listening and compassion, governance arrangements, and community empowerment, all of which were emphasized as key practices for creating change and generating impact.

Burgess (28) explains that academics who are successful at change making are driven 'by a passion greater than simply adding another item to your CV' (29), p. 12) and this was the case within our sample of prevention researchers. Participants were particularly passionate about ensuring their work was of a high standard and methodologically robust to foster change. This was important given that some methods (e.g., systems dynamic modeling) were considered innovative and relatively new to the prevention research context, therefore highlighting their long history as robust methods in other fields was necessary. Prior research indicates that conducting studies that are high quality while also communicating their strengths and weaknesses is key to having an impact by influencing policy (30, 31).

The need to communicate in a persuasive manner was emphasized within our sample. This meant learning the language of key stakeholders, adjusting communication styles to meet the needs of diverse groups be they senior government officials or community members, and avoiding jargon unless it was considered beneficial regarding technical use of terms and to ensure stakeholders were all on the same page. This aligns with the literature noting the importance of being able to adapt communication styles and formats to ensure relevance and comprehension (23). Something that was not however emphasized as much in our sample was the importance of storytelling for influencing policy, despite this being a well-documented necessity within the literature (32, 33). This omission is likely the result of our data collection tools not being specifically designed to explore leadership practices in depth.

Being able to situate research work within the policy and practice landscape was a key practice observed among participants. Instead of being focused first and foremost on situating research within an academic context, participants were primarily concerned with asking for, understanding, and then addressing the needs of communities and policymakers to ensure their work was impactful. Thus, demonstrating a keen awareness of policy processes, contexts, and stakeholder goals [as per (23)]. Being able to step into the shoes of key stakeholders, be they patients, community members, or policymakers, was an essential part of this process and was facilitated by compassion and deep listening practices. Being humble, approachable, and accessible to stakeholders involved in change processes served to build rapport and trust, which contributed to ongoing fruitful engagement and relationships [(as per (34, 35))], and enabled them to become embedded within the systems they sought to change. Self-awareness appears to be an important antecedent to the development of these important change making skills (36), along with the guidance of a trusted mentor who has already embodied these practices across their career and can provide honest feedback for reflection.

Oliver and Cairney (23) recommend that researchers should be clear on whether they are an advocate for particular topics or knowledge brokers presenting a more neutral position. We do note however that this may not always be a choice available for researchers due to funding agreement rules and restrictions. We observed a strong lean toward advocacy and activism within our sample, especially given their change making focus when it came to chronic disease prevention. Moreover, to be effective as change makers, participants spoke at length about the need to establish legitimacy and authority in their focus areas. When they were early in their careers or branching into new fields, they strategically teamed up with respected personnel or sought out mentors to help them achieve their goals. Oliver and Cairney (23) note the importance of being entrepreneurial or collaborating with someone who is able to act on their behalf. Owing to the benefit of having been mentored themselves, participants mentioned choosing to act as mentors for others to pass on skills and foster networks for those new to their field. They also emphasized the importance of capacity building both within their research teams and their stakeholder groups.

When it comes to addressing complex and systemic problems there are calls for leaders to cultivate practices that interact to create systems of leadership; these have been referred to as “collective' (7, 37), “shared” (38), “collaborative” (39), “emergent” (40), “co-leadership”, and “distributed” (11) leadership. Central to these concepts of leadership is the notion that leadership is a social process that is not reliant upon a single heroic leader but rather relies upon a systemic perspective of leadership whereby multiple actors take responsibility for change (10, 11). Distributed leadership, for example, calls for an emphasis on the attributes and behaviors of teams as opposed to individual leaders, while traditional forms of leadership typically focus on single leaders within organizations and systems (11). The topic of distributed leadership was not identified within our discussions with participants, however, this was likely a result of the interview guide not being geared toward this specific topic. This may be a fruitful area for further exploration in future prevention research studies exploring leadership and change making.

### Strengths and limitations

The findings reported here are qualitative, thus it is not possible to generalize the data collected and analyzed beyond the scope of the participants in the study. However, the information generated provides important insights that can assist prevention researchers to hone their leadership practices to foster greater impact in their work. Future research directions could explore a more explicit link between systems leadership practices and change making successes as well as how we can nurture systems leadership practices best suited to the prevention research workforce. Furthermore, future work could explore how leadership practices are distributed throughout teams and interact with the broader system and explore how the system supports or hinders their impact. Finally, we acknowledge that leadership is often a practice best judged from multiple perspectives within the system, therefore this may provide a beneficial area for further inquiry.

### Conclusion

While we are not offering a prescription for becoming a systems leader, we are suggesting that prevention researchers reflect upon the set of practices we have distilled in this paper by considering their natural strengths and then setting about to enhance these. Drucker (41) suggests that there is little to be gained by developing weaknesses as these will be the natural strengths of others; instead, the most fruitful gains can be made by building upon pre-existing strengths. In alignment with the systems literature and (39) work, we recommend steering away from the idea of a single heroic leader with a myriad of systems leadership traits and instead advocate for the creation of interdependent systems of leadership within chronic disease prevention research whereby leadership practices are developed and distributed throughout teams [as per (10, 11)].

## Data availability statement

The datasets presented in this article are not readily available because as per our ethics approval conditions, we are unable to share datasets outside of our research team. Requests to access the datasets should be directed to https://preventioncentre.org.au/.

## Ethics statement

This study involving human participants was reviewed by the Australian National University Human Research Ethics Committee-Ref No 2019/653. The participants provided their written informed consent to participate in this study.

## Author contributions

Conceptualization, methodology, and project administration: MP and LR. Formal analysis: MP, LR, MI, and EH. Funding acquisition: LR, SA, SF, and MP. Investigation: MI, EH, DF, TR, RI, and HR. Supervision: LR and SF. Writing—original draft: MP, LR, and MI. Writing—review & editing: MP, LR, SA, MI, EH, DF, TR, RI, HR, and SF. All authors contributed to the article and approved the submitted version.

## Funding

This study received funding from The Australian Prevention Partnership Center which was supported through the NHMRC partnership center grant scheme (Grant ID: GNT9100003) with the Australian Government Department of Health, ACT Health, Cancer Council Australia, NSW Ministry of Health, Wellbeing SA, Tasmanian Department of Health, and VicHealth. It is administered by the Sax Institute. The funder was not involved in the study design, collection, analysis, interpretation of data, the writing of this article, and the decision to submit it for publication.

## Conflict of interest

Authors MP, LR, MI, and EH were employed by The Australian Prevention Partnership Center. Author TR was employed by Therese Riley Consulting. The remaining authors declare that the research was conducted in the absence of any commercial or financial relationships that could be construed as a potential conflict of interest.

## Publisher's note

All claims expressed in this article are solely those of the authors and do not necessarily represent those of their affiliated organizations, or those of the publisher, the editors and the reviewers. Any product that may be evaluated in this article, or claim that may be made by its manufacturer, is not guaranteed or endorsed by the publisher.
